# The Prognostic Value of Transcutaneous Oxygen Pressure (TcPO_2_) in Diabetic Foot Ulcer Healing: A Protocol for a Systematic Review

**DOI:** 10.3390/diagnostics15070909

**Published:** 2025-04-02

**Authors:** Andrea Bordonado-Murcia, Javier Marco-Lledó, Pilar Nieto-Gil, Luz Marina Zuluaga-Ríos, Paloma López-Ros, Irene Hernández-Martínez, David Montoro-Cremades, Jonatan García-Campos

**Affiliations:** 1 Private Practice, 03170 Rojales, Spain; andreabm.podologia@gmail.com; 2 Department of Behavioral Sciences and Health, Miguel Hernandez University, 03550 Sant Joan, Spain; jmarco@umh.es (J.M.-L.); plopez@umh.es (P.L.-R.); jgarcia@umh.es (J.G.-C.); 3 Institute for Health and Biomedical Research (ISABIAL), 03010 Alicante, Spain; 4 Facultad de Enfermería y Podología, Universidad de Valencia, 46010 Valencia, Spain; pilar.nieto@uv.es; 5 Hospital San Juan de Dios, 760044 Cali, Colombia; draluzmarinazuluaga@gmail.com; 6 Hospital IMED Elche, 03203 Elche, Spain; irene.her.mar@gmail.com

**Keywords:** prognosis, transcutaneous, blood gas monitoring, diabetic foot, foot ulcer

## Abstract

**Background/Objectives:** Due to poor perfusion, diabetic foot ulcers (DFUs) create hypoxic environments, and their chronicity represents a negative factor in wound healing. Transcutaneous oxygen pressure (TcPO_2_) is a non-invasive method that provides information on oxygen supply to microvascular circulation, useful for determining the severity and progression of peripheral arterial disease (PAD) as well as potentially predicting DFU healing. However, the current literature does not provide strong support for the use of TcPO_2_ as an independent predictive tool. **Methods:** This protocol aims to systematically review the available evidence according to PRISMA (2020) guidelines, registered with the International Prospective Register of Systematic Reviews (registration number: CRD42024505907). The following databases will be used: Cochrane Library, EMBASE, Ovid Medline, PubMed, and Web of Science. Additionally, a manual search will be conducted through the references of the included articles. **Results:** The systematic review will summarize the current evidence on the prognostic value of TcPO_2_ in DFU healing, identifying gaps in knowledge and potential areas for future research. **Conclusions:** The findings of this study may clarify the prognostic value of TcPO_2_ in DFU healing, which could ultimately facilitate clinical management, decision-making, patient care, and potentially reduce treatment costs.

## 1. Introduction

Diabetes mellitus (DM) is a chronic and progressive pathological condition that occurs when high blood glucose levels are persistently maintained due to a deficiency in the synthesis of the hormone insulin or significant alterations in its action [1]. This condition affects millions of people worldwide, and it is estimated that by 2030, the number of people affected by DM will rise to 578 million, and by 2045, this number will reach an alarming 700 million [2]. This constantly increasing trend highlights the urgent and imperative need to develop and implement better strategies for prevention, treatment, and awareness to effectively address and combat the expanding global diabetes epidemic.

There are various complications associated with DM, such as macrovascular diabetic complications, including diabetic coronary artery disease; microvascular diabetic complications, such as diabetic nephropathy and diabetic retinopathy; diabetic neuropathy, including peripheral neuropathy and autonomic neuropathy; as well as other conditions like periodontal disease, diabetic bone diseases, and diabetes-related skin disorders, among others [3]. One of the most common complications of DM is diabetic foot ulcer (DFU). DFU is defined as the infection, ulceration, or destruction of the tissues of the foot of a person with previously diagnosed DM, frequently accompanied by neuropathy and/or peripheral arterial disease of the lower limb [4]. The incidence rate of DFUs can reach up to 34%, with a recurrence rate of up to 40% within one year [5,6]. On the other hand, the delay in the diagnosis of DM, as well as the management of the disease itself and the various associated complications associated with it, imply a significant socio-health economic expense, with a considerable increase in recent decades [1].

DFU is frequently accompanied by peripheral neuropathy (PN), defined as the presence of symptoms or signs of peripheral nerve dysfunction, and/or peripheral arterial disease (PAD), defined as an obstructive atherosclerotic disease of the arteries from the distal aorta to the foot, with clinical symptoms, signs, or abnormalities on non-invasive or invasive vascular testing or medical imaging. These conditions result in disturbed or impaired circulation in one or both of the lower extremities [7]. Both entities are strongly related to a worse prognosis in ulcer healing, increased risk of amputation, and higher risk of mortality [8], making early diagnosis and management essential.

PAD affects approximately 50% of patients with DFUs, significantly complicating the wound healing process. This condition negatively impacts healing, leading to a considerably increased risk of amputation and mortality [6,9]. Due to poor perfusion, hypoxic environments limit tissue recovery in DFUs [10]. Although hypoxia initially stimulates the wound healing process, its chronic persistence becomes a negative factor, inhibiting proper healing [11]. Establishing a prognosis for wound healing is essential in the management of DFUs, with local microcirculation and tissue oxygenation being relevant factors for this purpose [12,13].

Unlike methods based on hemodynamic indices, such as the ankle–brachial index, plethysmography, or Doppler systolic pressure, the measurement of transcutaneous oxygen pressure (TcPO_2_) is a metabolic test. TcPO_2_ is a non-invasive method widely used in vascular medicine, particularly in patients with critical limb ischemia. This technique provides information on oxygen supply to the microvascular circulation by recording the partial pressure of oxygen on the skin surface, making it useful for determining the severity and clinical progression of PAD [14]. It is considered the gold standard for predicting DFU healing [12,13]. The technique has proven to be easily manageable by both experienced healthcare professionals and beginners, with an average reading time of approximately 35 min for the area of interest. The equipment acquisition costs are affordable for a typical hospital in developed countries, and the operational costs are considered negligible [15]. Additionally, this test is applicable to all patients, regardless of Doppler signals, pulse palpation, or the presence of painful lesions [16]. The test is performed by applying a sensor to the skin using a flat, double-sided adhesive ring. Oxygen diffuses according to its pressure gradient (PaO_2_) from the capillary loops through the avascular epidermis to the electrode on the skin surface [17]. When PaO_2_ is normal, there is a direct relationship between TcPO_2_ values and local perfusion levels. Therefore, a decrease in PaO_2_ leads to a reduction in TcPO_2_ measurements [18]. This makes TcPO_2_ an important indicator of tissue perfusion and oxygenation, which are critical factors in wound healing and overall patient prognosis.

Although new diagnostic tools and techniques, such as TcPO_2_, are constantly being developed, it is crucial to thoroughly assess their accuracy and critically evaluate the existing literature before they are integrated into clinical practice. Although TcPO_2_ has been widely studied as a predictor of DFU healing, the available evidence presents methodological limitations, and no consensus exists on its reference values [19]. Moreover, the literature supporting the use of TcPO_2_ as an independent predictive tool remains limited in methodological quality. To date, no systematic review has rigorously synthesized the available data to establish standardized clinical criteria. This knowledge gap represents a critical scientific issue that must be addressed to enhance the clinical application of TcPO_2_ and improve therapeutic decision-making. Achieving consensus on reference values would be a pivotal step in optimizing its clinical utility. Furthermore, defining the precise relationship between TcPO_2_ levels and DFU healing prognosis would mark a significant advancement, enabling cost reductions in treatment and providing both patients and clinicians with more reliable prognostic information. Ultimately, this could lead to improved decision-making and better patient outcomes.

A systematic review protocol is crucial as it allows for the careful review, planning, and anticipation of potential issues, documents their approach before starting, and enables other researchers to compare the protocol with the final review. It ensures transparency, helps replicate methods, prevents arbitrary decisions on inclusion criteria and data extraction, and reduces the duplication of efforts while promoting collaboration [20]. We hypothesize that TcPO_2_ is a reliable predictor of DFU healing and that specific threshold values can be identified to enhance clinical decision-making. Despite its widespread use, there is no consensus on the optimal TcPO_2_ cut-off values for predicting wound healing. Previous studies present methodological limitations, highlighting the need for a structured and transparent approach to synthesize the available evidence. Therefore, this systematic review protocol aims to define a rigorous methodology to assess the prognostic value of TcPO_2_ in DFU healing. Specifically, it establishes a strategy for evaluating the quality of existing studies, identifying optimal TcPO_2_ thresholds, and developing evidence-based recommendations to improve clinical decision-making and therapeutic strategies.

## 2. Experimental Design

### 2.1. Research Design

To thoroughly synthesize existing evidence on TcPO_2_ values and the prognosis of DFU healing, and to ensure that the systematic review is conducted and reported with accuracy and clarity, we have developed a protocol following the PRISMA-P (preferred reporting items for systematic review and meta-analysis protocols) statement [20]. The PRISMA-P checklist contains 17 numbered items divided into three main sections: administrative information, introduction, and methods. The PRISMA-P checklist is harmonized with PRISMA (preferred reporting items for systematic reviews and meta-analyses) checklist items [21].

### 2.2. Protocol Registration

This protocol has been registered with the International Prospective Register of Systematic Reviews (PROSPERO: CRD42024505907).

### 2.3. Inclusion Criteria for Study Selection

**Figure 1:**
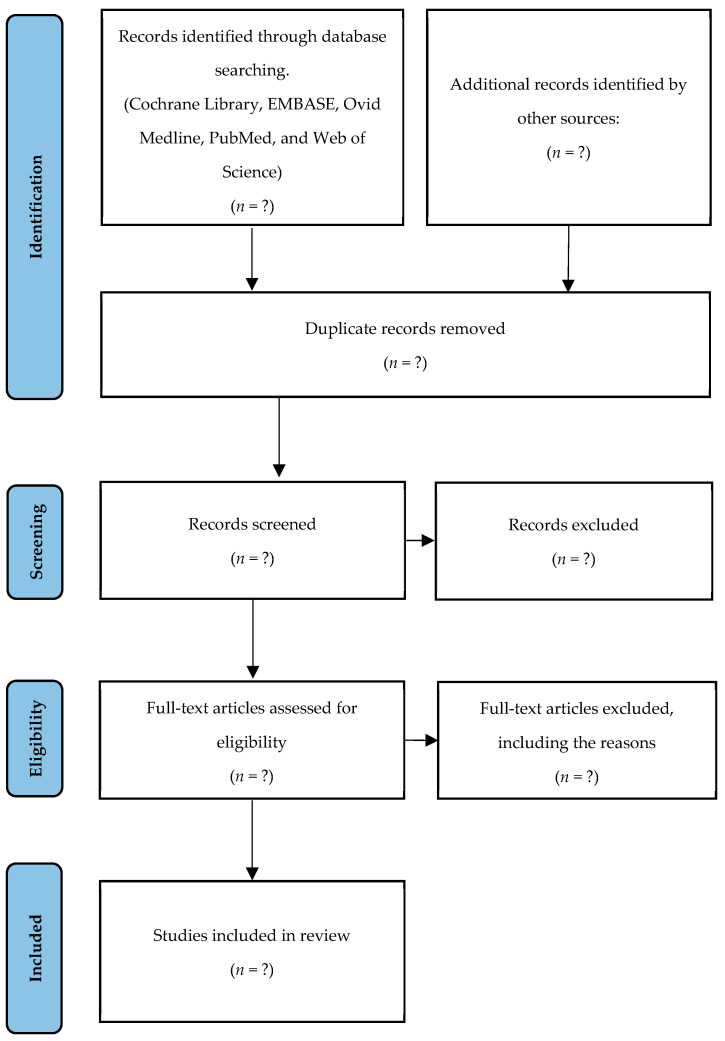
A PRISMA flow diagram of the study selection process.

A systematic approach is employed to ensure the inclusion of high-quality and relevant studies while minimizing potential biases. The study selection process follows the PRISMA guidelines [21], which provide a structured framework for transparently reporting the identification, screening, eligibility assessment, and inclusion of studies. This methodology enhances consistency, reliability, and credibility in the study selection and data analysis (Figure 1).

#### Types of Studies

Randomized Controlled Trials and Cohort Studies assess the efficacy of interventions in controlled settings. Case-Control Studies observe outcomes over time in groups of patients with varying TcPO_2_ levels and compare healed and non-healed patients based on their TcPO_2_ levels. Diagnostic Test Validation Studies verify the accuracy and reliability of TcPO_2_ as a diagnostic tool for predicting DFU healing outcomes.

The review is limited to studies published in English and Spanish. There are no limitations on publication status or publication date. Certain types of studies, including editorials, commentaries, opinions, study protocols, and literature reviews, are expected to be ineligible for inclusion. Furthermore, publications that do not directly align with the review’s objectives or do not meet predefined quality criteria are excluded. Potential exclusions involve studies with incomplete or inaccessible data. Additionally, preliminary investigations, publications lacking rigorous peer-review processes, or those primarily relying on self-reported data without verification are also excluded.

Studies including participants aged 18 and older diagnosed with DM and having at least one DFU are included, without limitations regarding sex, race, educational level, disease duration, previous treatments, or other comorbidities.

Studies that use TcPO_2_ measurement as a screening tool in individuals with DFU and DM are included.

The primary outcome measure is the TcPO_2_ measurement value as a reference in the healing process of DFU in individuals with DM. Secondary outcomes include specific data related to prognosis such as healing rates and the time to healing.

## 3. Materials and Methods

A preliminary scoping search was conducted in PROSPERO and the Cochrane Library to ensure that this objective had not been addressed by previous studies. The search strategy includes the following databases: Cochrane Library, EMBASE, Ovid Medline, PubMed, and Web of Science. To ensure objectivity and minimize bias, all searches will be conducted independently by two reviewers across various electronic databases, following the systematic review protocol. Additionally, reference lists of relevant articles are manually reviewed, and academic search engines, such as Google Scholar, are used to identify additional publications from the grey literature.

A sensitive search strategy is designed using relevant search terms derived from Medical Subject Headings (MeSH), the collection of free-text terms (TIAB), and keywords generated from the topic headings: “Blood Gas Monitoring, Transcutaneous” [Mesh], “Prognosis” [Mesh], “Wound Healing” [Mesh], “Foot Ulcer” [Mesh], “Diabetic Foot” [Mesh]. Additionally, a manual search is conducted through the reference lists of the included articles. Eligible studies are included up to April 2025. All search strategies employed in the study are meticulously recorded. The detailed search strategy in the different databases is available in Appendix A.

### 3.1. Study Selection and Data Extraction

#### 3.1.1. Study Selection

All studies identified by the search strategy are screened using the eligibility criteria. Initially, duplicates are removed. To achieve this, the retrieved records are exported to bibliographic management software. It is recommended to use the software’s automatic duplicate detection features to identify and eliminate duplicate records. Furthermore, a manual review is conducted to ensure the removal of any remaining duplicates, thereby ensuring a clean and duplicate-free set of records. This combined approach of automatic and manual checks helps guarantee that no irrelevant or repetitive studies are included, improving the overall accuracy and integrity of the systematic review process.

Documents are selected based on the titles and abstracts of the identified articles, which are meticulously assessed to identify potentially relevant publications. To determine eligibility, a thorough examination of the full texts of these selected articles is required. The entire identification and selection process is conducted independently by at least two researchers. In cases where discrepancies arise, a third reviewer resolves the differences to reach a consensus decision.

#### 3.1.2. Data Extraction

**Figure 2:**
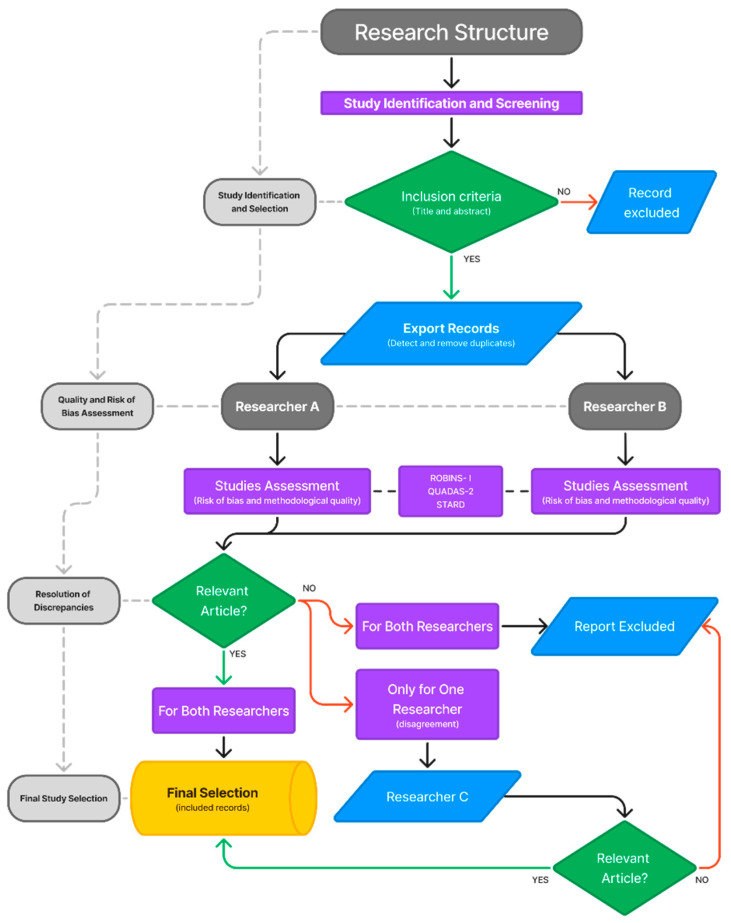
A flowchart of the study selection and assessment process. The figure outlines the methodological strategy, starting from the literature analysis and inclusion criteria, followed by the independent study assessment by two reviewers using the ROBINS-I, QUADAS-2, and STARD tools. Studies that meet the inclusion criteria proceed to the quality assessment, while excluded records are documented. In case of disagreement, a third reviewer evaluates the study to reach a final decision on inclusion.

Following the study selection, two reviewers independently extract data using a predefined standard data extraction form that includes general information such as author; year published; country of publication; study design; sample size and its characteristics; health professionals involved; the accuracy and reliability of the screening tool; intervention; outcomes; and adverse events. In cases of disagreements regarding data extraction, the issue is discussed and resolved by a third reviewer. This structured process ensures consistency, accuracy, and thoroughness in collecting essential study details for analysis. The overall methodology, encompassing literature screening, inclusion criteria, and quality assessment, is summarized in Figure 2.

Studies are categorized based on their primary and secondary objectives. A clear delineation of primary objectives is crucial for focusing on the core theme, while secondary objectives refine the scientific emphasis further. Parameters of interest for analysis include sensitivity, specificity, and positive and negative predictive values. These data are organized and presented in a descriptive summary reflecting their original depiction in the studies.

The rigorous assessment tools QUADAS-2 [22], ROBINS-I [23], and the STARD checklist [24], in conjunction with the dual-reviewer approach, guarantee that the chosen diagnostic accuracy studies maintain stringent criteria for quality and relevance.

The QUADAS-2 tool (Quality Assessment of Diagnostic Accuracy Studies) [22] is designed to assess the quality and risk of bias in diagnostic accuracy studies, making it a versatile instrument for evaluating diagnostic accuracy across various areas of medicine. It is primarily used in systematic reviews to assess the reliability of studies evaluating diagnostic tests, ensuring that the test results are accurate and applicable to clinical practice. QUADAS-2 is structured around four key domains of diagnostic accuracy studies: participant selection (which assesses whether the selection process introduced bias), diagnostic tests (which examines how the tests were conducted and whether they were implemented properly), reference standard (which analyzes whether the standard used to determine the final diagnosis was appropriate), and patient flow and timing (which evaluates the temporal sequence of the tests and the follow-up of patients in the study). Evaluators answer specific questions in each domain to determine whether the risk of bias is high, low, or unclear. Each domain is evaluated for its potential to introduce bias that could affect the study’s results, and the extent to which the study’s findings can be applied to clinical practice is also assessed, considering whether the study design and conditions are comparable to real-world scenarios.

ROBINS-I (Risk Of Bias In Non-randomized Studies—of Interventions) [23] is a tool designed to assess the risk of bias in non-randomized studies of interventions. These types of studies, also known as observational or non-randomized studies, refer to those in which participants are not randomly assigned to intervention or control groups, which may introduce biases that affect the results and conclusions. Before assessing specific bias domains, ROBINS-I includes a preliminary stage in which key considerations are evaluated to ensure a rigorous risk-of-bias assessment. This phase involves identifying relevant confounding factors, clearly defining the study’s research question in alignment with the systematic review, and determining whether the study is a randomized trial (in which case ROBINS-I is not applicable, and ROB-2 should be used instead). Additionally, for non-randomized studies, this stage assesses whether appropriate statistical methods were used to estimate the effect of interest, including adjustments for confounding factors. Once these preliminary considerations are addressed, the tool evaluates seven key domains that can introduce bias in non-randomized studies: (1) bias due to confounding, which occurs when external factors influence the relationship between the intervention and the outcome; (2) bias in the selection of participants, which arises if inclusion/exclusion criteria favor certain groups, affecting comparability; (3) bias in the classification of interventions, which occurs if intervention and control groups are not correctly defined or if misclassification takes place; (4) bias due to deviations from intended interventions, which emerges when there are differences between the planned and received intervention or if group management varies; (5) bias due to missing outcome data, which arises when data loss during follow-up could alter the results; (6) bias in the measurement of outcomes, which happens if outcomes are not measured objectively or if the assessor is aware of patient allocation; and (7) bias in the selection of reported outcomes, which occurs when researchers selectively report only certain outcomes that favor their hypothesis. Each domain is classified as low risk, moderate risk, high risk, or critical risk, based on the severity of the bias. The results of ROBINS-I can be summarized using different approaches, such as summary tables, which list each included study and its risk of bias classification across the seven domains; traffic light plots, which visually represent bias levels using green (low risk), yellow (moderate risk), red (high risk), and dark red (critical risk); or narrative summaries, which describe trends in bias risk across studies, highlighting domains with a consistently low or high risk.

The STARD checklist (Standards for Reporting of Diagnostic Accuracy Studies) [24] is a structured guideline designed to enhance the quality and transparency in the reporting of diagnostic accuracy studies. Its purpose is to ensure that studies report all relevant aspects of diagnostic test accuracy in a complete and clear manner, facilitating the interpretation and replication of results and helping to identify well-designed, reliable studies. This contributes to better clinical decision-making and the improvement of evidence-based practice. The checklist includes 30 items covering all stages of the study, from design to data analysis. In this way, it promotes a detailed description of all aspects of the study, from patient selection to how diagnostic tests were conducted, allowing other researchers to replicate the study or assess its quality and applicability. By standardizing reporting, the STARD checklist facilitates comparison between different diagnostic studies, contributing to a more robust and reliable body of evidence.

### 3.2. Risk of Bias

Systematic reviews of diagnostic accuracy studies often exhibit diverse findings due to differences in study design and execution. Therefore, a meticulous evaluation of the quality of the included studies is essential.

Stringent objectivity must be upheld throughout the process. Each publication should undergo an independent assessment by two reviewers. This dual-review method is implemented to guarantee that all decisions are based on comprehensive evaluations and remain impartial. In cases where discrepancies occur during selection, a third reviewer should be consulted to achieve consensus, thereby maintaining the integrity of the selection process.

The risk of bias and methodological quality of the included articles are assessed independently using specific tools tailored to each study design. The Revised Tool for Assessing Risk of Bias in Randomized Trials will be applied to RCTs, the Risk Of Bias In Non-Randomized Studies of Interventions (ROBINS-I) [23] to cohort and case-control studies, and the Quality Assessment of Diagnostic Accuracy Studies (QUADAS-2) [22] to Diagnostic Test Validation Studies. The STARD checklist [24] is utilized for reporting diagnostic accuracy studies to enhance completeness and transparency. All assessment tools undergo pilot testing to ensure consensus among reviewers. Any discrepancies are resolved by involving a third reviewer.

To further ensure the validity of the review findings, meta-biases are assessed, specifically evaluating whether published studies exhibit a tendency to report positive results over negative outcomes. This step aims to mitigate the potential influence of publication bias on the overall synthesis of evidence.

### 3.3. Strength of Evidence

**Figure 3:**
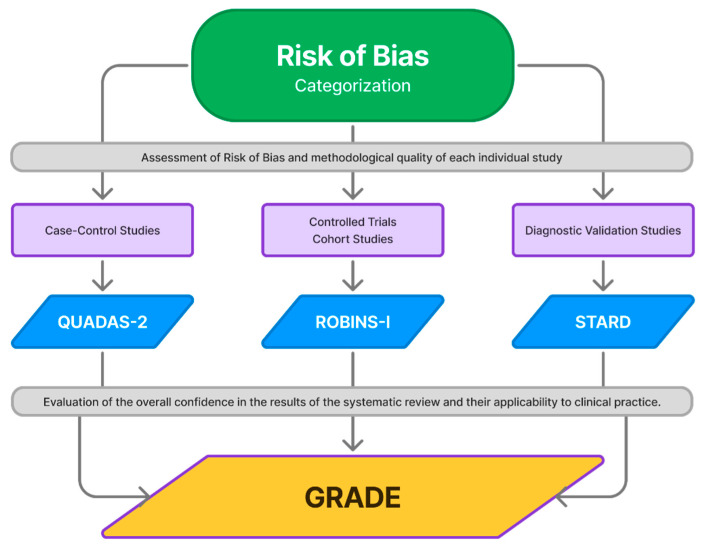
A flowchart representation of the GRADE Approach in the Systematic Review Process. This diagram illustrates the sequential assessment of the included studies based on their design, applying ROBINS-I, STARD, or QUADAS-2 accordingly. Once individual studies are evaluated for risk of bias and methodological quality, the GRADE framework is used to assess the overall certainty of the evidence. GRADE considers key factors such as risk of bias, inconsistency, indirectness, imprecision, and publication bias to determine the confidence in the review’s conclusions. This final evaluation ensures the clinical applicability of the findings, supporting evidence-based decision-making.

The quality of the evidence is evaluated using the Grading of Recommendations, Assessment, Development, and Evaluation (GRADE) framework (Figure 3). This system categorizes evidence into four levels: high, moderate, low, and very low quality. The GRADE approach ensures a systematic and transparent process for assessing the certainty of evidence, providing a robust foundation for drawing conclusions and making recommendations.

## 4. Discussion

Local microcirculation and tissue oxygenation are relevant factors in the prognosis of DFU healing [12,13]. These factors can be quantified by TcPO_2_, a non-invasive method that provides information on oxygen delivery to the microvascular circulation, useful in determining the severity and progression of PAD [14]. However, there is little literature with adequate methodology to support the use of TcPO_2_ as an independent predictive tool [19].

Systematic reviews provide one of the highest levels of scientific evidence, as they pool and critically evaluate all available studies on a topic. This review would allow such robust evidence to be generated, consolidating the role of TcPO_2_ in clinical decision-making. Although TcPO_2_ measurements as a prognostic tool for DFU healing can provide valuable information, their reliability and applicability in current studies may be influenced by several factors, including measurement variability, patient and ulcer characteristics, and sample size, among others. The review by Forsythe et al. [25] suggests developing protocols to standardize data collection and help better determine which demographic and clinical factors predict healing failure.

Recent studies reported different thresholds and predictive values for DFU healing using TcPO_2_. The study by Yang et al. [26], included 61 diabetic patients with foot ulcers. Participants were classified into three groups according to their healing outcome: healed ulcer, partial healing, and failed to heal or their ulcer deteriorated. This study found that a TcPO_2_ threshold of 25 mmHg is the optimal value for predicting diabetic foot ulcer healing. It was identified that patients with TcPO_2_ ≤ 10 mmHg did not improve, while those with TcPO_2_ ≥ 40 mmHg achieved complete wound closure. Furthermore, Lopez-Moral et al. [27] established that TcPO_2_ was the best predictor of diabetic ulcer healing compared to other non-invasive techniques. However, this study was not without limitations. Furthermore, the study by Fagher et al. [28], which included 236 diabetic patients with DFUs and a mean age of 76 years, found that a TcPO_2_ < 25 mmHg was associated with increased 1-year mortality, whereas the anklebrachial index (ABI) did not show a significant influence on mortality.

On the other hand, a systematic review published in 2020 [29], identified that factors related to equipment handling may influence measurement variations, such as correct electrode location and application, electrode temperature, and the use of a reference electrode. TcPO_2_ readings can vary significantly depending on the placement of the electrode for measurement, as shown in the study by López-Moral et al. [30], which highlights the different sensitivities and specificities depending on the angiosome tested. In the angiosome dorsalis pedis (dorsum of the foot), a sensitivity of 95% and a specificity of 73% was obtained for ulcers located in the forefoot, while placing the electrode in the posterior tibial angiosome, a sensitivity of 100% and a specificity of 85% was achieved for ulcers located in the midfoot and heel. The mean intra-individual variation is 8 mmHg, suggesting that variations within this range may not be clinically significant [31]. The variability depending on the measurement site raises questions about its reliability in clinical decision-making. Some studies suggest that alternative methods, such as FLIR thermography, may provide complementary information on limb perfusion [32].

Similarly, factors such as temperature, wound dimensions, and the time of evolution can also affect TcPO_2_ readings, complicating the interpretation of results [26,33]. Other confounding factors that may affect TcPO_2_ measurement include the presence of comorbidities such as hypertension, renal involvement, the presence of osteomyelitis, severe oedema or heart failure [27], age, the presence of amputation, or the level of amputation [34].

Although TcPO_2_ has been shown to be more reliable than other tests such as the ankle–brachial index (ABI) or finger blood pressure, there is no consensus on which test is the best predictor of ulcer healing. Some studies highlight that TcPO_2_ has a higher specificity and sensitivity than ABI, but the magnitude of this superiority varies between studies [28].

Some of the drawbacks related to this measurement technique that we have encountered refer to studies that have used populations with different clinical characteristics to measure TcPO_2_. The study by Kalani et al. [13] included 50 diabetic patients with a mean age of 61 years with chronic foot ulcers and a mean duration of diabetes of 26 years. This work evaluated healing using TcPO_2_, dividing patients into three groups according to clinical outcome: impaired ulcer healing, improved ulcer healing, and healed with intact skin. This study found that a TcPO_2_ < 25 mmHg was associated with a poor prognosis for healing.

TcPO_2_ is a non-invasive test that has gained popularity; however, its routine use is not yet fully established in all clinical settings. Determining the relationship between TcPO_2_ values and the prognosis of DFU healing would be a significant clinical advance in the use of this test. Therefore, a critical evaluation is needed to assess the available evidence through a systematic review of the existing literature with the aim of synthesizing the available evidence regarding the prognostic factor of TcPO_2_ and its relationship with the predictive ability of DFU healing, facilitating clinical management and decision-making, reducing treatment costs, and providing more accurate information on disease progression [35,36].

It is also important to consider that variability in TcPO_2_ results may be attributed to technical and human factors. The calibration of the equipment, the experience of the operator, and adherence to standardized protocols are crucial for obtaining accurate and reproducible measurements. The lack of standardization in TcPO_2_ measurement methodology across different studies complicates the comparability of results and limits the generalization of conclusions.

In summary, although TcPO_2_ shows great potential as a predictive tool in the healing of diabetic foot ulcers, it is essential to address the limitations and variabilities associated with its use. The standardization of measurement protocols, consideration of confounding factors, and integration of TcPO_2_ with other diagnostic techniques may enhance its reliability and clinical applicability. A thorough and well-designed systematic review can provide the necessary evidence to consolidate the role of TcPO_2_ in clinical decision-making and improve outcomes for patients with diabetic foot ulcers.

## 5. Limitations

This systematic review protocol presents certain methodological limitations that should be acknowledged. One potential concern is publication bias, as studies with positive results are more likely to be published, potentially affecting the representativeness of the findings. Additionally, the restrictiveness of the inclusion and exclusion criteria, determined by the specific characteristics of the TcPO_2_ technique and its role in DFU healing, could limit the identification of relevant studies, impacting both the comprehensiveness of the review and the generalizability of its conclusions. Another limitation is the language restriction, as only studies published in English and Spanish will be considered. This decision, while ensuring methodological rigor, may inadvertently exclude relevant evidence available in other languages, which could influence the overall synthesis. Lastly, the time required to conduct the systematic review poses a challenge, as new studies published during or after its completion might alter the existing evidence base, potentially affecting the conclusions.

## 6. Conclusions

Currently, the available literature does not provide strong support for the use of TcPO_2_ as an independent predictive tool of DFU healing. Additionally, there is no consensus on the specific values of this test for its interpretation. Therefore, it is essential to conduct rigorous research, establishing precise and well-defined measurement procedures.

### Recommendations

This research protocol outlines a standardized approach to systematically review the methodology regarding the utility of TcPO_2_ as a predictive tool for DFU healing. The methodology aims to uphold rigorousness, transparency, and reproducibility, placing emphasis on robust evaluation methods such as the QUADAS-2 [22], ROBINS-I [23], and STARD Checklist [24] for diagnostic accuracy studies. This protocol is intended to serve as a guide for future researchers undertaking similar review studies, employing specific tools to support each phase of the study process. The findings will be synthesized to provide a comprehensive evaluation of the available evidence, guiding both the potential integration of TcPO_2_ measurements into clinical practice and the identification of key areas for future research.

## Data Availability

The original contributions presented in this study are included in the article. Further inquiries can be directed to the corresponding author.

## Abbreviations

DM—Diabetes Mellitus; DFU—Diabetic Foot Ulcer; PAD—Peripheral Arterial Disease; TcPO_2_—Transcutaneous Oxygen Pressure; RCTs—Randomized Controlled Trials; QUADAS-2—Quality Assessment of Diagnostic Accuracy Studies; ROBINS-I—Risk Of Bias In Non-randomized Studies of Interventions; ABI—Ankle–Brachial Index.

## Appendix A. Search Strategy by Database

PubMed
(“Blood Gas Monitoring, Transcutaneous”[Mesh] OR “Blood Gas Monitoring, Transcutaneous”[TIAB]) AND (“Prognosis”[Mesh] OR “Prognosis”[TIAB]) AND (“Wound Healing”[Mesh] OR “Wound Healing”[TIAB]) AND ((“Foot Ulcer”[Mesh] OR “Foot Ulcer”[TIAB]) OR (“Diabetic Foot”[Mesh] OR “Diabetic Foot”[TIAB]))
MEDLINE Ovid
(Blood Gas Monitoring, Transcutaneous.sh. or Blood Gas Monitoring, Transcutaneous.ab. or Blood Gas Monitoring, Transcutaneous.ti.) and (Prognosis.sh. or Prognosis.ab. or Prognosis.ti.) and (Wound Healing.sh. or Wound Healing.ab. or Wound Healing.ti.) and (Foot Ulcer.sh. or Foot Ulcer.ab. or Foot Ulcer.ti. or (Diabetic Foot.sh. or Diabetic Foot.ab. or Diabetic Foot.ti.))
Web of Science
(TI=(Blood Gas Monitoring, Transcutaneous) OR AB=(Blood Gas Monitoring, Transcutaneous)) AND (TI=(Prognosis) OR AB=(Prognosis)) AND (TI=(Wound Healing) OR AB=(Wound Healing)) AND (TI=(Foot Ulcer) OR AB=(Foot Ulcer)) AND (TI=(Diabetic Foot) OR AB=(Diabetic Foot))
Cochrane Library
((Blood Gas Monitoring, Transcutaneous) AND (Prognosis) AND (Wound Healing) AND ((Foot Ulcer) OR (Diabetic Foot))):ti,ab,kw
EMBASE
(’transcutaneous oxygen monitoring’/exp OR ’transcutaneous oxygen monitoring’ OR (transcutaneous AND (’oxygen’/exp OR oxygen) AND (’monitoring’/exp OR monitoring)) OR ’transcutaneous oxygen monitoring’,ti) AND (’prognosis’/exp OR prognosis,ti) AND (’wound healing’/exp OR ’wound healing’ OR ((’wound’/exp OR wound) AND (’healing’/exp OR healing)) OR ’wound healing’,ti) AND (’foot ulcer’/exp OR ’diabetic foot’/exp OR ’diabetic foot’,ti)
